# Bone tunnel enlargement on anterior cruciate ligament reconstruction

**DOI:** 10.1590/1413-78522014220500338

**Published:** 2014

**Authors:** Adriano Barros de Aguiar Leonardi, Aires Duarte, Nilson Roberto Severino

**Affiliations:** 1 Santa Casa de São Paulo, Faculdade de Ciências Médicas, São Paulo, SP, Brazil, Faculdade de Ciências Médicas da Santa Casa de São Paulo, Pavilhão "Fernandinho Simonsen" São Paulo, SP, Brazil

**Keywords:** Anterior cruciate ligament/surgery, Tibia, Tendons, Femur, Muscles, Reconstructive surgical procedures

## Abstract

**Objective:**

To assess the presence of tibial bone tunnel enlargement after surgical reconstruction of the anterior cruciate ligament using quadruple graft of the flexor tendons and correlate the functional results in their presence.

**Methods:**

The studied lasted six months and included 25 patients, with ages ranging from 18 to 43 years old. Assessment was based on radiographs taken immediately postoperatively and at the third and sixth month of follow up in the anterior cruciate ligament reconstruction. Reconstruction of ligaments was performed with tendon grafts of the semitendinosus and gracilis muscle fixated in the femur with transverse metal screw and in the tibia with interference screws. Patients were evaluated objectively by tests ligament, graded from zero to four crosses and subjectively by the Lysholm method preoperative and after sixth month follow up.

**Results:**

Significant increase in the tunnels diameters were observed, 20.56% for radiographs in the anteroposterior view, 26.48% in profile view and 23.22% in computed tomography. Descriptive statistics showed significant improvement in subjective and objective clinical parameters.

**Conclusions:**

The bone tunnel enlargement is a phenomenon found in the first months after surgical reconstruction of the anterior cruciate ligament and it has no implications on clinical outcomes in the short term. *Level of Evidence II, Prospective Study.*

## INTRODUCTION

Enlargement of bone tunnels after reconstruction of the anterior cruciate ligament has been a well-documented phenomenon in the literature since the early 90s. It is characterized by the enlargement of the tibial and femoral tunnels in X-rays and other postoperative sequential imaging tests.^1^ Its incidence is extremely variable, from 0% to 74.26%^2^ and it is closely linked to factors such as graft fixation and the method of measurement used. For knees operated with hamstring, rates of enlargement vary between 11% and 73.9% compared to 2.1% and 47%^3^ for those aged 3 patellar tendon. Taking into account the distance of fixing the articular surface, the rates vary from 0% to 23% for grafts submitted to anatomic fixation^4^ and 47% to 73.9% for those fixated distant from the articular surface.^3^

Although many studies report its occurrence, none proved to be clinically significant, or related to surgical failure rates.^1^^,^ 4 ^-^ 7 Its mechanism is also not yet fully understood. Possible causes are mechanical factors such as mobility of the graft in the tunnel, stress located at the entrance of the tunnel, improper positioning of the tunnels and aggressive rehabilitation.^1^^,^ 2 ^,^ 8 Biological factors include nonspecific inflammatory response mediated by cytokines, cell necrosis by toxic products (ethylene oxide, metal), immune response to foreign bodies (autologous grafts) and cellular necrosis in response to bone drilling.^3^

Statistically, it would have greater incidence on tibial tunnel^9^ and despite short and long term studies^10^ did not correlate its occurrence to surgical failures, concerns exist in cases where a surgical revision would be necessary.

The objectives of this study are to assess the presence of the tibial bone tunnel enlargement after surgical reconstruction of the anterior cruciate ligament using quadruple grafting of flexor tendons and to correlate functional outcomes in their presence.

## PATIENTS AND METHODS

In a prospective study, we followed up 25 patients who underwent surgery for reconstruction of the anterior cruciate ligament by video-arthroscopic technique with quadruple graft of semitendinosus and gracilis tendons fixed by transverse metal screws TransFix^(r)^ on the femur and tibia interference, being 23 male (92%) and two females (8%). The mean age of the group was 28.5 years old, ranging from 18 to 43 years old, 16 patients having been operated the right side (64%) and nine patients the left side (36%); the average time span between the occurrence of the injury and surgery was 9.2 months, ranging between three and 25 months. All patients were operated by the Department of Knee Surgery CEMKA (Kawano Medical Center) at *Hospital Nossa Senhora do Rosário* of Intermédica network, in São Paulo, SP, Brazil. The study was conducted from June 2006 to August 2008 after approval by the Ethics Research Committee.

The criteria for inclusion of patients in the study were the following: a) complete rupture of the anterior cruciate ligament diagnosed clinically and by nuclear magnetic resonance imaging (MRI); b) complaints of instability; c) other knee ligaments intact; d) articular cartilage intact; e) no lesion of the posterior meniscus horn; f) absence of neurological and vascular injury or previous fractures of the lower limbs; g) no alteration of unilateral load axis; h) not presenting previous injuries of the locomotor system that lead to functional limitation, articular amplitude limitation or changes in muscle function; i) not having been submitted to previous knee surgery on the knee to be operated. All patients signed a Free and Informed Consent Form.

### Postoperative follow-up

To collect data, we standardized the following periods:

a) T0 - Preoperative period;
b) T1 - Postoperative period from 0 to 30 days;
c) T2 - 3 months postoperative period;
d) T3 - 3 months postoperative period.

Clinical evaluation was performed for objective parameters were made on the surgical table, always by the same surgeon with the patient subjected to spinal cord lock. Included the Lachman test anterior drawer in neutral position and the pivot shift, quantified progressively from zero to four crosses. The same tests were repeated on the sixth postoperative month.

For subjective parameters the Lysholm^11^ scale was used, ranging from 0 to 100 points and ranks the results as "excellent" 95-100 points; "Good" 84-94 points; "Regular" 65-93 points and Poor, when lower than 64 points.

**Figure 1:**
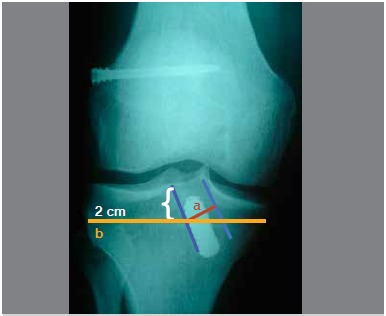
Anteroposterior radiography (AP). The blue line follows the edges of the tunnel and at 2 cm below the medial joint line, perpendicular thereto, its diameter is represented by the line "a". At the same point the diameter of the bone is calculated, represented by line "b". The value obtained by the variable "a" is then divided by the constant.

**Figura 2:**
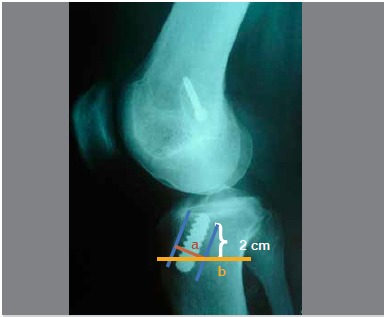
Profile radiography (P). A) The blue line follows the edges of the tunnel and at 2 cm below the medial joint line, perpendicular thereto, its diameter is represented by the line "a". At the same point the diameter of the bone is calculated, represented by line "b". The value obtained by the variable "a" is then divided by the constant .

Radiographs of the operated knees of each patient were performed in the postoperative period, the anteroposterior (AP) and lateral (P) views. (Figures 1 and 2) The first X-Ray was taken in the immediate postoperative period (T1) after skin closure and dressing; and the remaining at the third (T2) and sixth (T3) months after surgery. The diameter of the tunnel in the tibia was measured at 2.0 cm below the articular line of the medial tibial condyle from the sclerotic margins of the visible path of the drill and, by drawing a line perpendicular to the tunnel, generating the variable "a". The values obtained were divided by the diameter of the bone, generating the constant "b" also measured at 2.0 cm below the medial joint line. The option to generate relative results given by the ratio a/b was taken in order to avoid the possible biased results by magnification of the radiograph.

The nonparametric Wilcoxon test for comparison of the variables found in the proportion a/b on radiographs (AP1, AP2, AP3, P1, P2 and P3) with respect to various times (T0, T1, T2 and T3) was used.

In all tests, the significance level of 5% was used, the tests being statistically significant with p <0.05. The results considered statistically significant were indicated by an asterisk (*), and the not significant by n.s.

## RESULTS

Of the thirty patients selected for the study, three were excluded for not attending the pre-determined appointments and two for having poor imaging records, being impossible to measure the diameters of the tunnels.

There wasn't, in any case, complications such as infection, deep vein thrombosis and nerve injury.

All patients completed the postoperative rehabilitation in the expected period and there was no case of range of motion restriction.

Considering the criteria adopted for subjective assessment, we noticed that the preoperative mean of 24.12 points and 100% classified as "bad" increased to 95.72% and 72 classified as "excellent", 24% as "good" and 4% as "regular" in the postoperative period. (Tables 1 and 2)

**Table 1:** Evolution of patients according to Lysholm criteria for T0 (preoperative) and T3 (6th month postoperative).

		Excellent	Good	Regular	Poor	**Total**
T0	n	0	0	0	25	25
	%	0%	0%	0%	100%	100.0%
T3	n	18	6	1	25	25
	%	72.0%	24.0%	4.0%	100.0%	100.0%

**Table 2:** Descriptive statistics of the values obtained in the subjective evaluation of Lysholm for T0 (preoperative) and T3 (6th months postoperatively).

**Time**	**Mean**	**Median**	**St. Dev.**	**Minimum**	**Maximum**
T0	24.1200	23.0000	6.2738	16.00	43.00
T3	95.7200	96.0000	4.9793	81.00	100.00

Wilcoxon test between T0 and T3; p<0,001*.

**Figure 3:**
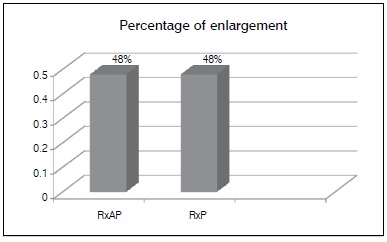
Incidence of the tibial bone tunnel enlargement in anteroposterior (RxAP) and profile (RxP) radiographs.

Considering the criteria adopted for objective assessment, we noticed that for the Lachman test, we observed in the preoperative period that 17 patients who had two crosses, four patients had three crosses and one patient four crosses. In the postoperative period, only one patient had three crosses and two patients with one cross. For the Anterior Drawer Test in neutral rotation, of the 19 patients who had two crosses, four patients, one cross and one patient four crosses in the postoperative period, we observed only one ranked with three crosses and two patients with one cross. For the pivot-shift test, of the 20 patients who had two crosses, two patients had three crosses and of patients with one cross in the postoperative period, we observed only one patient ranked with two crosses. (Table 3) Considering the enlargement of the bone tunnels, the increased diameter of the tibial tunnel in successive measurements, i.e. T3 > T2 > T1, we found the phenomenon in 48% of radiographs. (Figure 3)

**Table 3:** Evolution of patients according to Lachman test, posterior drawer test and pivot shift. The left column expresses the number of crosses found during the test and the upper, times T0 and T3.

		**T0**			**T3**			**Total**		
		**Lach**	**AD**	**PS**	**Lach**	**AD**	**PS**	**Lach**	**AD**	**PS**
**+1**	**n**	-	-	2	-	-	0	-	-	2
**+2**	**n**	17	19	20	2	2	1	19	21	21
**+3**	**n**	4	2	2	1	1	0	5	3	2
**+4**	**n**	1	1	-	0	0	-	1	1	-
**Total**	**n**	22	22	24	3	3	1	25	25	25

Lach: Lachman test; AD: anterior drawer test; PS: Pivot-shift test.

**Figure 4:**
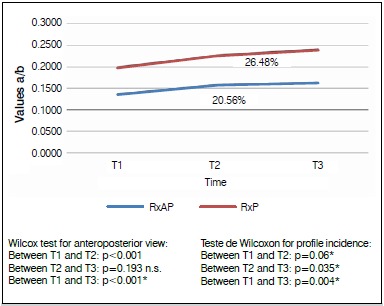
Progression of enlargement of the bone tunnels through the relative values a/b. RX AP and RX P represent, respectively, the anteroposterior and profile radiographs.

Using the measurement method proposed, we observed an increase of 16.37% for radiographs in the anteroposterior view in the period from T1 to T2 and 20.56% in the period from T2 to T3. In profile view radiographs, we found an increase of 16.99% for the period from T1 to T2 and 26.48% for the period from T2 to T3. (Figure 4)

## DISCUSSION

Enlargement of bone tunnels in reconstruction of the anterior cruciate ligament is a phenomenon that has been reported in recent years, regardless of the technique used.^1^^,^ 7 ^,^ 10 ^,^ 12

In the literature, there is no consensus regarding the method for its measurement. Some authors state that it should be measured in the sclerotic margins in the larger dimension of the bone tunnel, perpendicular to the long axis on postoperative knee radiographs.^8^^,^ 13 Other authors are in favor of measuring always at a certain point below the articular interline^5^ and at different points along the tunnels, due to the fact that it best describes the morphology of enlargement: conical, linear or cavitary.^14^ We also noticed that there is no consensus regarding the expression of results. While some authors do so in absolute numbers,^15^ others express enlargement in percentage.^3^^,^ 8 ^,^ 13

We chose Fahey and Indelicato's^16^ method and standardized a point 2 cm below the medial articular interline, where the phenomenon would occur with greater intensity.^1^^,^ 13 All scans were analyzed by the same examiner.

In order to obtain the radiographic magnification effect, where the image obtained in the films becomes greater than the studied object, some authors recommend that the correction factor is calculated and they estimate the calculated value to be up to 10% for conventional radiographs.^8^^,^ 16

In our study, we created a method where we obtained relative values between the diameter of the tunnel, which we call variable "a" and its long axis, which we call constant "b" (Figures 1 and 2), both measured at 2 cm below the articular interline. We believe that this method, in addition to preventing bone tunnels measurement errors due to the magnification of radiographs, also avoids errors when trying to establish the point of greatest diameter of the tunnel, because we believe it may lead to measuring at different locations on the same knee, and obviously, to erroneous results.

Regarding the time of emergence, it seems to be consensual that the enlargement of the tunnel may occur within the 1^st^ year after surgery, especially between the third and the ninth week, and may not occur, or occur in a very subtle way up to two to three postoperative years.^4^^,^ 10 We conducted the follow up for six months which, according to the literature, would be ideal for the phenomenon to occur and we noticed that it was significant and more intense in the first three months, and then less intense between three to six months, with statistical significance for the anteroposterior radiograph of the tibia and not statistically significant for the anteroposterior view of the tibia. (Figure 4)

The definition of the etiology of bone tunnel enlargement seems to be a great challenge and it is still unknown.^1^ Currently, two distinct lines of thought are used to explain its occurrence. The mechanical factors theory includes the mobility of the graft within the tunnel, asymmetrical stress inside the tunnel wall, improper graft placement, and aggressive rehabilitation. Authors that advocate this theory claim that micro movements generated within the bone tunnels would cause changes in the bone-graft integration and enlargement of the tunnels would be result of it.^1^ ^,4.5^ The theory that advocate biological factors is based on possible action of nonspecific inflammatory response mediated by cytokines, cell necrosis due to toxic products (ethylene oxide, metal), necrosis and heat in response to perforation.^2^

Studies comparing anatomical fixation, in which the interference screw fixation remains fair to articulate a distance, using the same graft, showed significantly higher rates of enlargement in the last^2^ compared fixation with Endobutton (Smith & Nephew Endoscopy, Andover, MA) in the femur and tibia Ethibond^(r)^ sutures with a bioabsorbable interference screws in both bone graft using hamstring tendons. They obtained rates of enlargement of 31% in the first group and 65% in the second and concluded that, since they have noticed bony enlargement after anatomic and stable fixation, only biological factors are linked to the genesis of the phenomenon. Faun and Kaalund,^15^ in a similar study, noticed enlargement in 12.29% and 43.47% of the tibial tunnels of operated knees with anatomic and distance fixation, respectively. Barber *et al*.^4^ compared patellar tendon grafts fixated with interference screws in a group the same with one corner folded over itself and noted the rate of 20% for the former and 0% for the second. They concluded that enlargement of the bone tunnels would be linked only to the micro-movement of the graft inside the tunnels, because once eliminated the tendon portion that is traditionally "left" between the interference screw and the exit of the tunnel, it would eliminate the effect of the "windshield wiper", and would reduce the enlargement of the bone tunnels.

Comparatively analyzing these authors' results, we find strong evidence that the viscoelastic properties of each graft may be linked to the presence of enlargement of the tunnels because there were different rates for knees fixated the anatomical way. We believe this is an indication in favor of the mechanical theory. However, we disagree with Barber *et al*.^4^ that the patellar tendon graft folded on itself eliminates the enlargement of bone tunnels for just reducing the micro-movement. Once the tunnel exit is sealed, it also eliminates the contact of the graft to the synovial fluid, with consequent reduction of cytokine action.

Some authors found significantly higher rates of enlargement of the tunnel where there was technical error in making the tunnels, especially when the tibial tunnels were made in a way prior to the conventional one, i.e., before the femur Blumensaat line on profile radiographs.^6^

Analyzing the results of these authors, we believe that this is the biggest clue in favor of the mechanical theory of enlargement of the bone tunnels, mainly on the micro-movement of the graft within the bone tunnel, because, according to the authors, an error in positioning them would lead to the impact against the intercondyle roof, limiting the knee extent.^6^^,^ 17 We believe this would cause abnormal forces on the graft and would be responsible for increased mobility in the tendon-bone interface, changes in its incorporation, stretching, and possible failure.

Rodeo *et al.*,^18^ in a biomechanics study, found a direct relationship between the mobility of the graft, distance from the attachment to the articular surface and osteoclastic activity. Their results are in agreement with those of Springer *et al.*,^19^ who noticed increased tension of the graft in the tibial tunnel entry, both in the sagittal and coronal plane, from 0 to 30 degrees of flexion and attribute to it the delay in wound healing and consequent enlargement of bone tunnels.

We believe that these papers clearly demonstrate the direct relationship between micro-movement of the graft and the

occurrence of enlargement of the bone tunnels. However, taking into account that the phenomenon is more intense in the first six months, and that after, it does not occur, or occurs in smaller rates,^10^^,^ 13 coupled with the fact we did not find any study that proves that this mobility is reduced or stops at the end of the integration of the graft to the bone tunnel, we do not believe that the micro-movement of the graft within the bone tunnel is an isolated factor in its genesis.

Some authors compared the relationship of enlargement of the bone tunnels with cytokine concentrations in samples of synovial fluid in the postoperative period. Zysk *et al*.^7^ studied knees operated with hamstring tendon and patellar tendon and noticed that in all cases where the phenomenon was present, there were increased concentrations of tumor necrosis factor alpha (αTNF), interleukin 6 (IL-6) and nitric oxide (NO). The authors proposed that the synovial fluid, rich in interleukins, irrigating the tendon-bone interface, an effect they termed "synovial bath", alter the biological fixation of the graft and would be linked to the genesis of the enlargement of the bone tunnels. But these authors did not notice differences of these compounds' concentrations among knees operated with different grafts.

Analyzing the study of these authors, we believe that there is a strong indication of the presence of biological factors, although the sample is small since only 13 patients were studied, however, they would not be enough to explain the phenomenon, since its occurrence was statistically higher in knees operated with hamstring graft, which leads us to believe that there are as much evidence of mechanical theory, as integration factors of each type of graft to the bone tunnel.

Healing and integration of tendons in bone tunnels are also pointed out as a possible factor linked to enlargement of bone tunnels. Studies by Rodeo *et al.* 18 indicate tissue similar to Sharpey fibers at around six weeks and performing resistance tests, there would be failure of tendon-bone interface up to twelve weeks. Thereafter, this failure would occur in the graft itself. According to the authors, there is a higher intensity of the inflammatory response mediated by lympho-monocytic infiltrate during the first eight weeks. The other would be marked by more subtle changes.

In our study, we observed a more intense enlargement and with statistical significance during the first nine weeks (Figure 4) during which, according Rodeo *et al.*,^18^ there would be an increased inflammatory response in the integration of the tendon to the bone tunnels. Although studies of Rodeo *et al*.^18^ have been performed in an experimental extra-articular model, the tendon not having been subjected to contact with synovial fluid and, thus, exposed to the possible effects of their proteolytic enzymes and the fact that they kept the tendon vascularized during the study, there was, thus, no necrosis, one of the stages that the graft would go through during its integration to the bone, we believe that it reflects the healing of the graft in the bone tunnels and points to a strong clue that it relates to the genesis of the bone tunnels enlargement.

Most studies analyzing the phenomenon of enlargement of the bone tunnels versus time correlated the presence of tunnel enlargement with clinical impairment and recurrence of instability,^10^^,^ 13 in a four years retrospective study, the correlation between bone tunnels enlargement and surgical failure was also not noticed.

In the present study, in agreement with these authors, we found no statistically significant difference between the clinical results and enlargement of the tunnel. Although we noticed that the enlargement of the bone tunnels is statistically significant for both radiographs in AP and profile views (Figure 4) and preset a 48% incidence of the patients studied, we also observed clinical improvement by analyzing objective (Tables 1 and 2) and subjective parameters. (Table 3) Although we observed that the phenomenon has clinical implications in the short term, we believe we it has the potential to cause problems with the positioning and fixation of the graft in revision surgery, and that the enlargement of the bone tunnels should, therefore, be avoided, when possible.

## CONCLUSIONS

Enlargement of bone tunnels is a phenomenon present in the first months after anterior cruciate ligament reconstruction surgery, and does not have implications in clinical short-term results.
